# Exertional Hyponatremia Among Active Component Members of the U.S. Armed Forces, 2008–2023

**Published:** 2024-04-20

**Authors:** Armed Forces Health Surveillance Division Defense Health Agency, US

## Abstract

Exertional hyponatremia, or exercise-associated hyponatremia, occurs within 24 hours after physical activity due to a serum, plasma, or blood sodium concentration (Na+) below the normal reference range of 135 mEq/L. If not detected early and managed properly, hyponatremia can be fatal. From 2008 to 2023, 1,812 cases of exertional hyponatremia were diagnosed among U.S. active component service members (ACSMs), with an overall incidence rate of 8.3 cases per 100,000 person-years (p-yrs). In 2023 there were 153 cases of exertional hyponatremia diagnosed among ACSMs, resulting in a crude incidence rate of 11.7 per 100,000 p-yrs. Female service members, those older than 40, non-Hispanic Black service members, Marine Corps members, recruits, those in combat-specific occupations, and ACSMs stationed in the Northeast U.S. region had higher incidence rates of exertional hyponatremia diagnoses than their respective counterparts. During the surveillance period, annual rates of incident exertional hyponatremia diagnoses peaked in 2010 (12.8 per 100,000 p-yrs) and then decreased to a low of 5.3 cases per 100,000 p-yrs in 2013. Thereafter the incidence rate fluctuated but has increased from 6.2 per 100,000 p-yrs in 2017 to its second-highest level in 2023. Service members and their supervisors should be aware of the dangers of excessive fluid consumption and prescribed limits for consumption during prolonged physical activity including field training exercises, personal fitness training, or recreational activities, particularly in hot, humid weather.

**What are the new findings?:**

The incidence rate of exertional hyponatremia in 2023 increased from 2022, reaching the second highest incidence rate since 2008.

**What is the impact on readiness and force health protection?:**

Exertional hyponatremia, which can be fatal if not promptly recognized and appropriately treated, has been increasing among U.S. service members over the past decade to a near record high, posing a significant health risk to U.S. military members. Military members, leaders, and trainers must be vigilant for early signs of hyponatremia, intervene immediately and appropriately, and observe the published guidelines for proper hydration during physical exertion, especially during warm weather conditions.

## 1 BACKGROUND

Exertional hyponatremia is a relatively rare disease that can be fatal if not detected early and managed properly. Exertional hyponatremia is caused by increased consumption of hypotonic fluids such as water or sports drinks before or during strenuous physical activity, including prolonged military field training or combat operations, or it can be caused by inappropriate secretion of a non-osmotic antidiuretic hormone due to physical exertion that results in increased total body water and free water retention.^1^ Hyponatremia is particularly problematic in the military, where it can be mistaken for heat exhaustion or heat stroke.

Active component military personnel, who often perform heavy physical activity in hot, remote, or austere environments, during training or under combat conditions, are particularly susceptible to fluid and electrolyte imbalances.^2,3^ Normal plasma sodium (Na+) concentration ranges from 135 to 145 mEq/L, and is closely regulated, along with osmolarity, to preserve cell size and function.^4^ Excessive intake of sodium stimulates thirst to increase body water to maintain serum (Na+).^5^ When a serum or plasma sodium concentration is less than 135 mEq/L within the 24 hour period following prolonged physical activity, hyponatremia or exercise-related hyponatremia occurs.^6^ There is growing evidence that hyponatremia is associated with increased morbidity, mortality, and health costs in a variety of clinical scenarios and diseases.^7^

The incidence of hyponatremia, resulting from a variety of activities including endurance competitions, hiking, police training, American football, fraternity hazing, and military exercises varies widely, is dependent upon activity duration, heat or cold stress, availability of water, and other individual risk factors. Other important risk factors besides excessive fluid intake include exercise duration of greater than 4 hours, inadequate training for an exertional event, and either high or low body mass index.^8^ Symptoms depend on the extent and rate of decrease in serum sodium compared to baseline levels. To reduce the risk of exertional hyponatremia, mitigation measures such as fluid and electrolyte replacement guidelines, identification of high-risk individuals, and vigilance during associated activities can be adopted.^9^

Exercising in hot weather continues to cause preventable injuries and deaths in young, healthy people.^3^ Considering the characteristics of military environments such as long-term military training and combat operations, exertional hyponatremia may continue to pose a health risk to U.S. military personnel, significantly reducing performance and combat effectiveness. This report summarizes the frequency, rates, trends, demographic, geographic location, and military characteristics of exertional hyponatremia cases among active component service members (ACSMs) from 2008 to 2023.

## 2 METHODS

The surveillance population for this report consisted of all ACSMs of the U.S. Army, Navy, Air Force, Marine Corps, Space Force, and Coast Guard who served at any time during the surveillance period, from January 1, 2008 to December 31, 2023. Due to the recent establishment of the Space Force, its personnel were categorized as Air Force for this analysis.^10^

All data used to determine incident exertional hyponatremia diagnoses were derived from records routinely collected and maintained in the Defense Medical Surveillance System (DMSS). These records document both ambulatory encounters and hospitalizations of ACSMs of the U.S. Armed Forces in fixed military and civilian (if reimbursed through the Military Health System [MHS]) treatment hospitals and clinics worldwide. In-theater diagnoses of hyponatremia were identified from medical records of service members deployed to Southwest Asia or the Middle East whose health care encounters were documented in the Theater Medical Data Store (TMDS).

A case of exertional hyponatremia was defined as 1) a hospitalization or ambulatory visit with a primary (first-listed) diagnosis of “hypo-osmolality and/or hyponatremia” (International Classification of Diseases, 9th and 10th revisions, ICD-9:276.1; ICD-10:E87.1) and no other illness or injury-specific diagnoses (ICD-9:001–999; ICD-10:A–U) in any diagnostic position or 2) both a diagnosis of “hypo-osmolality and/or hyponatremia” (ICD-9:276.1; ICD-10:E87.1) and at least 1 of the following within the first 3 diagnostic positions (dx1–dx3): “fluid overload” (ICD-9:276.9; ICD-10:E87.70, E87.79), “alteration of consciousness” (ICD-9:780.0*; ICD-10:R40.*), “convulsions” (ICD-9:780.39; ICD- 10:R56.9), “altered mental status” (ICD-9:780.97; ICD-10:R41.82), “effects of heat/light” (ICD-9:992.0–992.9; ICD-10:T67.0*–T67.9*), or “rhabdomyolysis” (ICD-9:728.88; ICD-10:M62.82).^11^

Medical encounters were not considered case-defining events if the associated records included the following diagnoses in any diagnostic position: alcohol or illicit drug abuse; psychosis, depression, or other major mental disorders; endocrine disorders; kidney diseases; intestinal infectious diseases; cancers; major traumatic injuries; or complications of medical care. An individual could be considered a case of exertional hyponatremia only once per calendar year. Incidence rates were calculated as cases of hyponatremia per 100,000 person-years (p-yrs) of active component service.

For health surveillance purposes, recruits were identified as active component members assigned to service-specific training locations during coincident service-specific basic training periods. Recruits were considered a separate category of enlisted service members in summaries of exertional hyponatremia by military grade overall.

In-theater diagnoses of exertional hyponatremia were analyzed separately using the same case-defining criteria and incidence rules used to identify incident cases at fixed treatment facilities. Records of medical evacuations from the U.S. Central Command (CENTCOM) area of responsibility (AOR) (i.e., Southwest Asia, Middle East) to a medical treatment facility outside the CENTCOM AOR were analyzed separately. Evacuations were considered case-defining if the affected service members met the aforementioned criteria in a permanent military medical facility in the U.S. or Europe from 5 days preceding until 10 days following their evacuation dates.

## 3 RESULTS

In 2023, there were 153 cases of exertional hyponatremia diagnosed among ACSMs, resulting in a crude incidence rate of 11.7 per 100,000 p-yrs, an increase from 8.8 per 100,000 p-yrs in 2022. From 2008 to 2023, there were 1,812 incident diagnoses of exertional hyponatremia among ACSMs resulting in a crude overall incidence rate of 8.3 cases per 100,000 p-yrs. **Table 1** presents the incident cases and incidence rates of exertional hyponatremia according to demographic characteristics.

In 2023, female ACSMs had a higher annual incidence rate (13.1 per 100,000 p-yrs) than males (11.4 per 100,000 p-yrs); a change from prior years when the rate was similar between sexes. Service members aged 40 and older had the highest incidence rate, followed by those less than 20 years of age (28.8 and 20.0 per 100,000 p-yrs, respectively). Non-Hispanic Black service members had the highest incidence rate (15.0 per 100,000 p-yrs) compared to other race and ethnicity categories. As with overall 2008-2023 rates, Marine Corps members had the highest incidence rate in 2023 (17.8 per 100,000 p-yrs) compared to other services.

There were 21 cases of exertional hyponatremia among recruits in 2023, an incidence rate 10 and nearly 6 times higher than those of other enlisted service members and officers, respectively. Combat-specific military occupations (infantry/artillery/combat engineering/armor) had the highest incidence rate (13.8 per 100,000 p-yrs) in 2023, excluding the other/unknown group.

The Northeast region of the U.S. had a higher incidence rate of exertional hyponatremia (14.6 per 100,000 p-yrs) compared to other regions in 2023.

**Figure 1:**
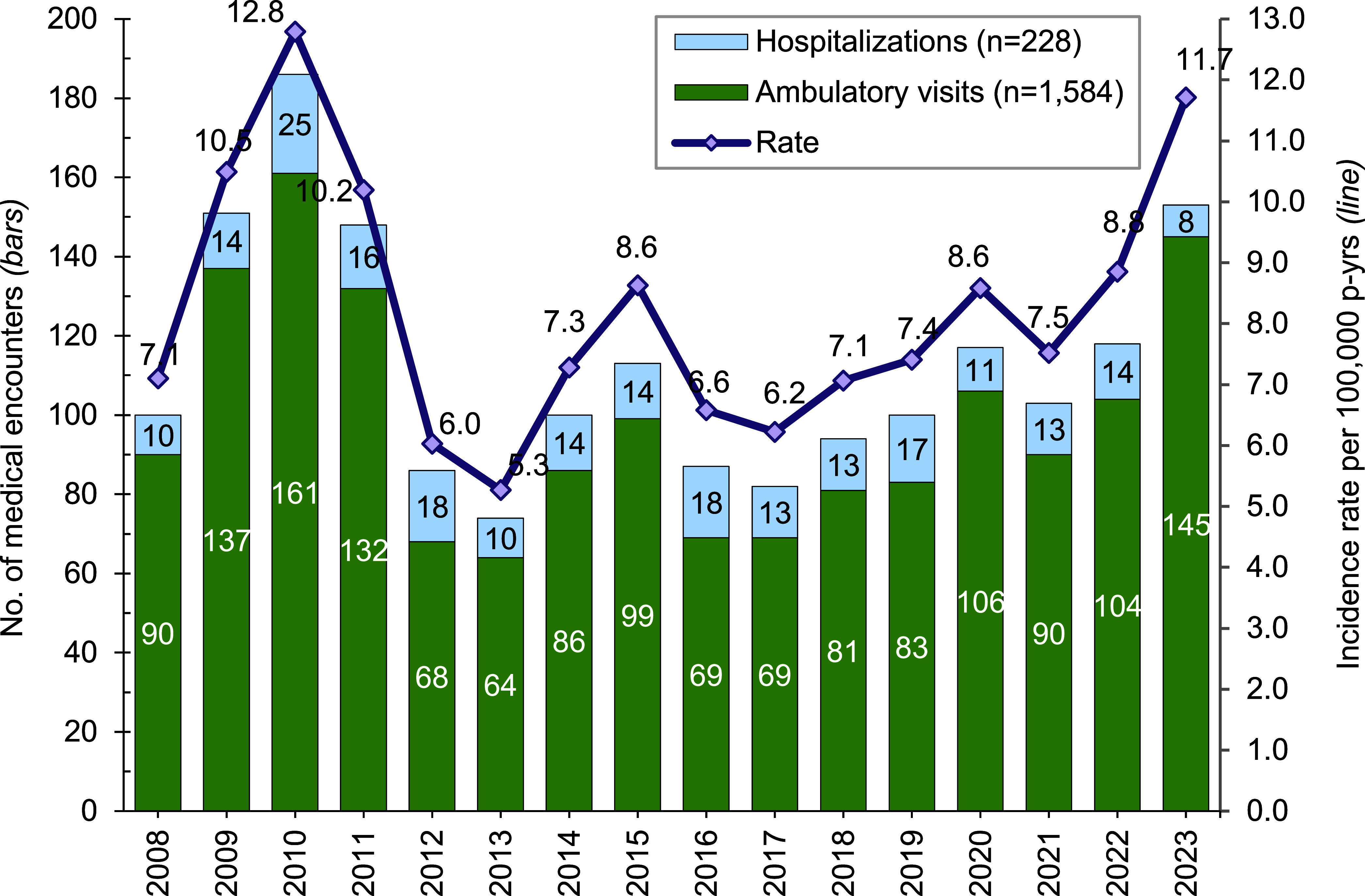
Annual Incident Cases and Rates of Exertional Hyponatremia, Active Component, U.S. Armed Forces, 2008-2023

**Figure 2:**
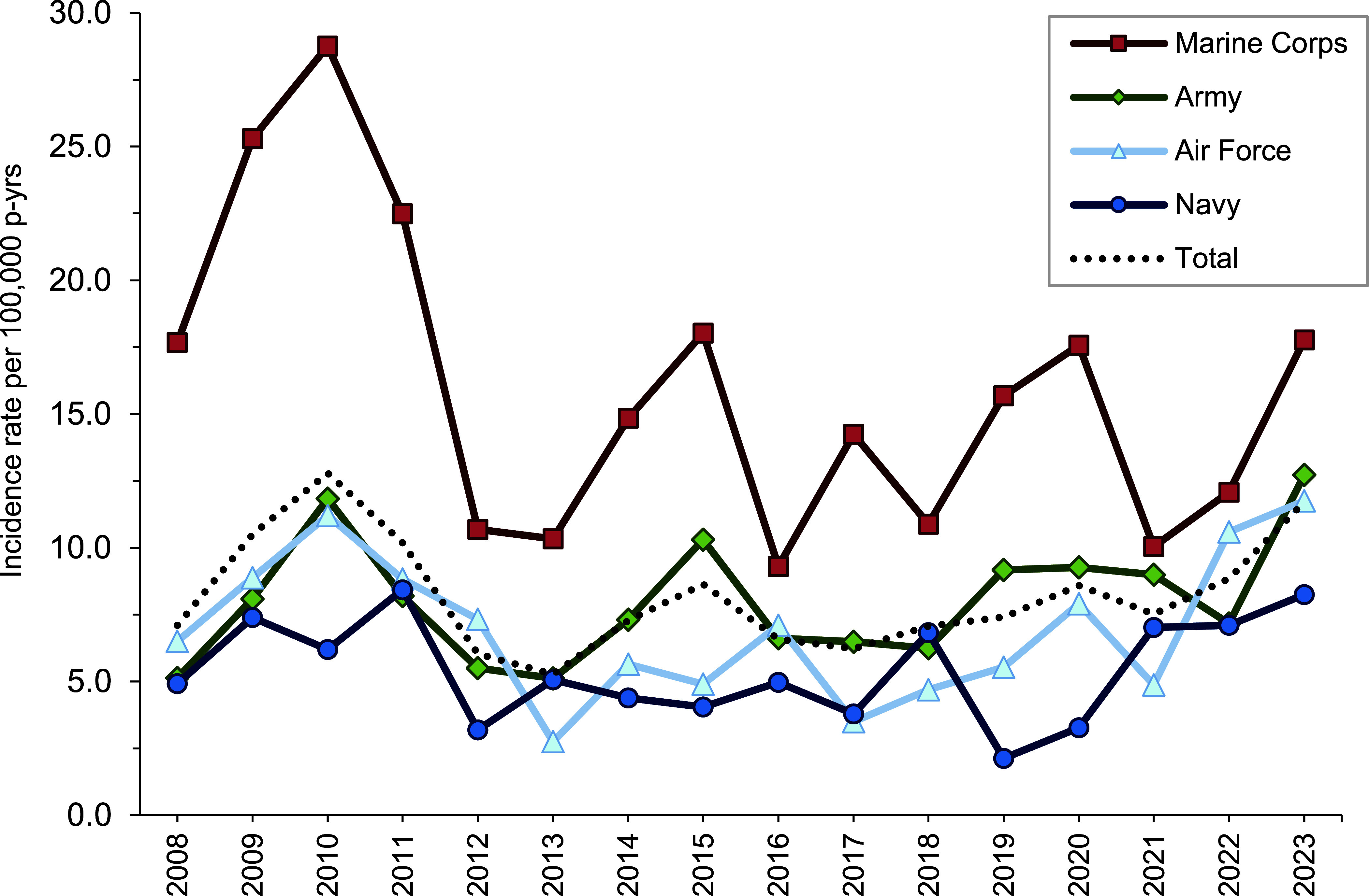
Annual Incidence Rates of Exertional Hyponatremia, by Service, Active Component, U.S. Armed Forces, 2008-2023

**Figure 1** presents annual incident cases and rates of exertional hyponatremia among ACSMs by year. Between 2008 and 2023, crude annual rates of incident exertional hyponatremia diagnoses peaked in 2010 (12.8 per 100,000 p-yrs) and then decreased to a low of 5.3 cases per 100,000 p-yrs in 2013. During the last 10 years of the surveillance period, rates fluctuated but generally increased from 6.2 cases per 100,000 p-yrs in 2013 to 11.7 cases per 100,000 p-yrs in 2023. The annual incidence of exertional hyponatremia diagnosis was significantly higher in the Marine Corps than in any other service (**Figure 2**). During the 16-year period, 87.4% (n=1,584) of all cases were diagnosed and treated without hospitalization (data not shown).

**Exertional hyponatremia by location**

During the 16-year surveillance period, exertional hyponatremia cases were diagnosed at more than 150 U.S. military installations and geographic locations worldwide, but 16 U.S. installations contributed 20 or more cases each and accounted for 49.2% of the total cases (**Table 2**). Marine Corps Recruit Depot (MCRD) Parris Island, SC, reported 203 cases of exertional hyponatremia, the highest in the DOD.

**Exertional hyponatremia in the CENTCOM AOR**

From 2008 to 2023, a total of 35 cases of exertional hyponatremia were diagnosed and treated in the CENTCOM AOR (data not shown). No new cases were diagnosed in 2023. Deployed service members affected by exertional hyponatremia were most frequently male (n=27; 77%), 20–24 years old (n=16; 46%), non-Hispanic White (n=26; 74%), in the Army (n=19; 54%), enlisted (n=27; 77%), and in combat-specific (n=10; 29%) or communications/intelligence (n=10; 29%) occupations (data not shown). Five service members were medically evacuated from the CENTCOM AOR for exertional hyponatremia, all of which occurred between 2009 and 2018 (data not shown).

## 4 DISCUSSION

Over the past decade, incidence rates of exertional hyponatremia have fluctuated, while overall increasing from 6.2 to 11.7 per 100,000 p-yrs in 2023. The incidence of exertional hyponatremia fluctuated more in women than in men. Although reports on the association between gender and hyponatremia provide conflicting analyses,^12,13^ many studies report that gender is not a significant risk factor for hyponatremia.^14,15,16,17^ Further investigation and ongoing monitoring may be warranted, however, to effectively prevent exertional hyponatremia in women in particular.

In 2023 the highest age-specific incidence rate was among the 40 years and older age group, which is consistent with the literature, in which increasing age has been reported as a strong independent risk factor for both hyponatremia and hypernatremia.^14^ Considering the rapid incidence rate increase in this age group, investigation of the causes of this change, to understand how this condition occurs and how it can be prevented, is warranted.

In *MSMR* analyses before April 2018, in-theater cases included diagnoses of hypoosmolality and hyponatremia in any diagnostic position, but in 2018 case-defining criteria for inpatient and outpatient encounters were applied to in-theater encounters. As a result, the results of the in-theater analysis are not comparable to those presented in earlier *MSMR* updates.

Several important limitations should be considered when interpreting this analysis. First, there is no specific diagnostic code for exertional hyponatremia. This lack of specificity may result in the inclusion of some non-exertional cases of hyponatremia, overestimating the true rate. Consequently, the results of this analysis should be considered estimates of the actual incidence of symptomatic exertional hyponatremia from excessive water consumption among U.S. military members. In addition, the accuracy of estimated numbers, rates, trends, and correlates of risk depends on the completeness and accuracy of diagnoses that are documented in standardized records of relevant medical encounters. As a result, an increase in recorded diagnoses indicating exertional hyponatremia may reflect, at least in part, increasing awareness, concern, and aggressive management by military supervisors and primary health care providers of incipient cases.

Finally, recruits were identified using an algorithm based on age, rank, location, and time in service, which was only an approximation and likely resulted in some misclassification of recruit training status.

Exertional hyponatremia must be differentiated from heat illness to avoid inappropriate treatment and adverse outcomes and instead, based on accurately observed signs and symptoms, appropriately diagnose and treat the condition. Well-trained personnel should be able to recognize signs of possible hyponatremia: excessive fluid intake, changes in mental status, vomiting, poor eating habits, abdominal bloating, and large amounts of clear urine.^3,9,18^

Considering the increased incidence rate of exertional hyponatremia in 2023, continued emphasis should be placed on how to effectively manage exertional hyponatremia, including prevention, identification, and treatment methods through close monitoring. Hyponatremia is treated primarily by managing the underlying cause in addition to free water restriction,^19^ concentrating on pre-hospital care through rapid on-site emergency medical service assessment in addition to hospital management in emergency and inpatient settings.^20^ Depending on the physical demands of military operations and prevailing environmental conditions, the composition of replacement fluids may vary.^21^

Due to the variety of underlying causes, individualized management may be the best approach to prevent exertional hyponatremia. Effective and collaborative management consistent with current policy and guidance to commanders is crucial for prevention of exertional hyponatremia (**Table 3**).^9^ It is critical to recognize exertional hyponatremia, provide appropriate treatment, and emphasize the importance of appropriate hydration practices to ensure service members’ health and performance.

## Figures and Tables

**Table 1:** Incident Cases^a^ and Incidence Rates^b^ of Exertional Hyponatremia, Active Component, U.S. Armed Forces, 2008-2023

	**2023**		**Total 2008-2023**	
	No.	Rate^b^	No.	Rate^b^
**Total**	153	11.7	1,812	8.3
** Sex**						
Male	123	11.4	1,524	8.2
Female	30	13.1	288	8.4
** Age group**, y						
<20	16	20.0	226	16.0
20-24	33	8.3	501	7.2
25-29	24	7.8	346	6.6
30-34	24	11.0	231	6.6
35-39	17	10.1	214	8.2
40+	39	28.8	294	12.8
** Race and ethnicity**						
White, non-Hispanic	75	10.9	1,153	9.0
Black, non-Hispanic	31	15.0	239	6.9
Hispanic	30	12.0	216	6.7
Other/unknown	17	10.6	204	8.1
** Service**						
Army	57	12.7	637	8.0
Navy	27	8.3	283	5.4
Air Force	38	11.8	362	7.0
Marine Corps	30	17.8	483	16.1
Coast Guard	1	2.6	47	7.5
** Military status**						
Enlisted	93	8.9	1,117	6.3
Officer	39	16.0	408	10.6
Recruit	21	90.2	287	64.4
** Military occupation**						
Combat-specific^c^	23	13.8	311	10.2
Motor transport	2	4.6	37	5.2
Pilot/air crew	4	8.8	46	5.8
Repair/engineering	33	8.9	328	5.1
Communications/intelligence	29	10.4	316	6.7
Health care	11	10.4	140	7.5
Other/unknown	51	17.3	634	14.5

Abbreviations: No., number; y, years.

^a^One case per person per year.

^b^Rate per 100,000 person-years.

^c^Infantry/artillery/combat engineering/armor.

**Table 2:** Incident Cases of Exertional Hyponatremia by Installation (with 20 cases minimum), Active Component, U.S. Armed Forces, 2008–2023

**Location of Diagnosis**	**No.**	**% Total**
MCRD Parris Island, SC	203	11.2
Fort Moore, GA	140	7.7
JB San Antonio, TX	68	3.8
Fort Liberty, NC	61	3.4
MCB Camp Lejeune/Cherry Point, NC	59	3.3
Walter Reed NMMC, MD^a^	37	2.0
NMC San Diego, CA	50	2.8
MCB Camp Pendleton, CA	45	2.5
NMC Portsmouth, VA	44	2.4
Fort Cavazos, TX	32	1.8
MCB Quantico, VA	31	1.7
Fort Campbell, KY	30	1.7
Fort Shafter, HI	26	1.4
Fort Carson, CO	22	1.2
Fort Belvoir, VA	22	1.2
Fort Jackson, SC	22	1.2
Other/unknown locations	920	50.8
Total	1,812	100.0

Abbreviations: No., number; MCRD, Marine Corps Recruit Depot; JBSA Joint Base San Antonio; AFB Air Force Base; MCB, Marine Corps Base; NMMC, National Military Medical Center; NMC Naval Medical Center.

Note: Recruit training locations include MCRD Parris Island, JBSA-Lackland AFB, MCB Camp Lejeune/Cherry Point, MCB Camp Pendleton, and Fort Jackson. Referral centers include Walter Reed NMMC, NMC San Diego, and NMC Portsmouth.

**Table 3:** TRADOC Recommendations^a^ for Continuous Work Duration and Fluid Replacement in Warm and Hot Environments

		**Easy Work**		**Moderate Work**		**Heavy Work**		**Very Heavy Work**	
**Heat Category**	**WBGT Index (°F)**	**Work (min)**	**Water Intake (qt/hr)**	**Work (min)**	**Water Intake (qt/hr)**	**Work (min)**	**Water Intake (qt/hr)**	**Work (min)**	**Water Intake (qt/hr)**
1 (white)	78-81.9	NL^b^	1/2	NL^b^	3/4	110	3/4	45	3/4
2 (green)	82-84.9	NL^b^	1/2	NL^b^	1	70	1	40	1
3 (yellow)	85-87.9	NL^b^	3/4	NL^b^	1	60	1	25	1
4 (red)	88-89.9	NL^b^	3/4	180	1 1/4	50	1 1/4	20	1 1/4
5 (black)	>90	NL^b^	1	70	1 1/2	45	1 1/2	20	1 1/2

Notes: 1. Applies to average-sized and heat-acclimatized service member wearing the operational camouflage pattern uniform. 2. Fluid needs can vary based on individual differences (+/- 1/4 qt/hr) and exposure to full sun or shade (+/- 1/4 qt/hr). 3. CAUTION: Hourly fluid intake should not exceed 1 1/2 qts. 4. CAUTION: Daily fluid intake should not exceed 12 qts.

Abbreviations: TRADOC, Training and Doctrine Command; WBGT, wet bulb global temperature; F, Fahrenheit; min, minimum; qt, quart; hr, hour; NL, no limit.

^a^Reference 9, page 24.

No work limit per hour, up to 4 continuous hours.

## AUTHOR AFFILIATIONS

Defense Health Agency, U.S. Department of Defense
